# Supplementary figures and images for: Molecular detection of Leishmania donovani, Leishmania major, and Trypanosoma species in Sergentomyia squamipleuris sand flies from a visceral leishmaniasis focus in Merti sub-County, eastern Kenya
Source: Parasit Vectors. 2021 Jan 18;14:53. doi: 10.1186/s13071-020-04517-0 (PMC7812738; doi:10.1186/s13071-020-04517-0)

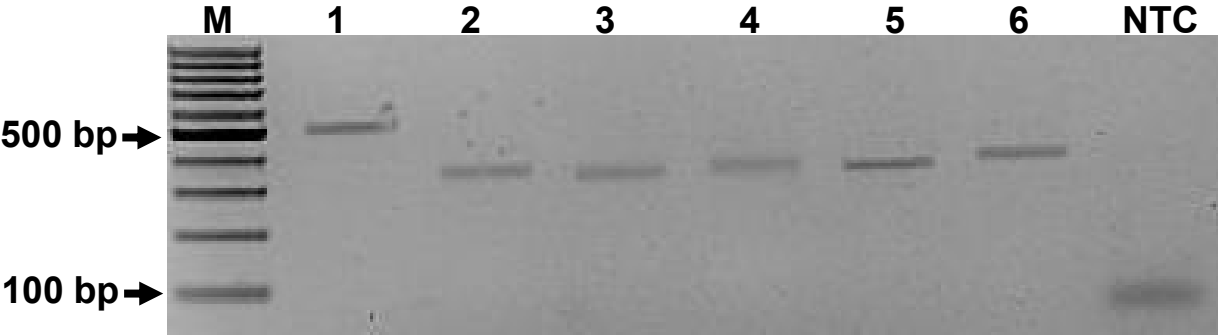

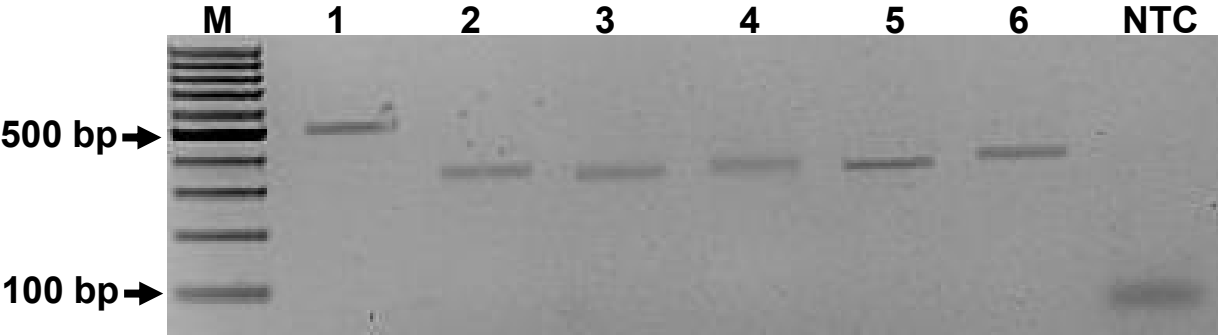

Supplement: Supplementary file 1 — Additional file 1: Figure S1. Detection of Leishmania and Trypanosoma spp. DNA in sand flies by ITS1-PCR. M: 100 bp ladder; 1–4: Sergentomyia squamipleuris sand fly samples; 5 and 6: Leishmania donovani (NLB065) and Leishmania major (Friedlin strain) positive controls; NTC negative control. [file 13071_2020_4517_MOESM1_ESM.pdf]
