# Supplementary material for: Molecular detection of Leishmania donovani, Leishmania major, and Trypanosoma species in Sergentomyia squamipleuris sand flies from a visceral leishmaniasis focus in Merti sub-County, eastern Kenya
Source: Parasit Vectors. 2021 Jan 18;14:53. doi: 10.1186/s13071-020-04517-0 (PMC7812738; doi:10.1186/s13071-020-04517-0)
Supplement: Supplementary file 2 — Additional file 2: Figure S2. Summary matrix of pair-wise patristic distances between the ITS1 sequences of Trypanosoma spp. identified in this study (red borders) and those registered in the GenBank. The patristic distances between a pair of sequences are represented in the form of a heatmap and their values indicated. The genetic distances were calculated in Geneious Prime (v20.0) following a maximum likelihood phylogenetic analysis implemented in PHYML. [file 13071_2020_4517_MOESM2_ESM.pdf]

	Trypanosoma spp. 269 (MT548851)	*T. dionisii* (JN040980.1)	*T. dionisii* (JN040978.1)	*T. cruzi* (JN040983.1)	*T. cruzi* (JN040982.1)	*T. lewisi* (HQ437158.1)	*T. avium* (AY959322.1)	*T. theileri* (JX178162.1)	*T. theileri* (JX853185.1)	*T. vivax* (KX584882.1)	*T. vivax* (KX584878.1)	*T. evansi* (LC199491.1)	*T. evansi* (LC199490.1)	*Bodo caudatus* (AY028450.1)	*T. grayi* (MG283141.1)	*T. grayi* (MG283146.1)	*T. rangeli* (AY230239.1)	*T. rangeli* (AY230238.1)	*T. congolense* (JN673388.1)	*T. congolense* (JN673389.1)	*T. brucei* (MK132171.1)	*T. brucei* (MK132169.1)
Trypanosoma spp. 269 (MT548851)		1.2	1.2	1.3	1.3	1.2	1.1	1.3	1.3	1.6	1.5	1.5	1.5	1.7	1.9	1.8	0.9	0.9	1.1	1.0	1.5	1.4
T. dionisii (JN040980.1)	1.2		0.1	0.9	0.9	1.1	1.1	1.3	1.3	1.6	1.5	1.5	1.5	1.6	1.8	1.8	1.1	1.2	1.2	1.2	1.4	1.4
T. dionisii (JN040978.1)	1.2	0.1		0.9	0.9	1.1	1.1	1.3	1.3	1.5	1.5	1.4	1.4	1.6	1.8	1.8	1.1	1.1	1.2	1.2	1.4	1.4
T. cruzi (JN040983.1)	1.3	0.9	0.9		0.3	1.2	1.1	1.3	1.3	1.6	1.6	1.5	1.5	1.7	1.9	1.9	1.2	1.2	1.3	1.2	1.5	1.5
T. cruzi (JN040982.1)	1.3	0.9	0.9	0.3		1.2	1.2	1.4	1.4	1.7	1.6	1.6	1.6	1.7	1.9	1.9	1.2	1.3	1.3	1.3	1.5	1.5
T. lewisi (HQ437158.1)	1.2	1.1	1.1	1.2	1.2		0.8	1.0	1.0	1.3	1.3	1.2	1.2	1.4	1.6	1.5	1.1	1.2	1.2	1.2	1.2	1.2
T. avium (AY959322.1)	1.1	1.1	1.1	1.1	1.2	0.8		0.4	0.4	0.8	0.8	0.6	0.6	0.9	1.1	1.1	1.0	1.1	1.1	1.1	0.8	0.8
T. theileri (JX178162.1)	1.3	1.3	1.3	1.3	1.4	1.0	0.4		0.0	1.0	1.0	0.7	0.7	1.1	1.3	1.3	1.2	1.3	1.3	1.3	1.0	1.0
T. theileri (JX853185.1)	1.3	1.3	1.3	1.3	1.4	1.0	0.4	0.0		1.0	1.0	0.7	0.7	1.1	1.3	1.3	1.2	1.3	1.3	1.3	1.0	1.0
T. vivax (KX584882.1)	1.6	1.6	1.5	1.6	1.7	1.3	0.8	1.0	1.0		0.2	1.2	1.2	0.9	1.1	1.1	1.5	1.6	1.6	1.6	1.3	1.3
T. vivax (KX584878.1)	1.5	1.5	1.5	1.6	1.6	1.3	0.8	1.0	1.0	0.2		1.2	1.2	0.8	1.0	1.0	1.5	1.5	1.6	1.5	1.2	1.2
T. evansi (LC199491.1)	1.5	1.5	1.4	1.5	1.6	1.2	0.6	0.7	0.7	1.2	1.2		0.0	1.3	1.5	1.5	1.4	1.5	1.5	1.5	1.2	1.2
T. evansi (LC199490.1)	1.5	1.5	1.4	1.5	1.6	1.2	0.6	0.7	0.7	1.2	1.2	0.0		1.3	1.5	1.5	1.4	1.5	1.5	1.5	1.2	1.2
Bodo caudatus (AY028450.1)	1.7	1.6	1.6	1.7	1.7	1.4	0.9	1.1	1.1	0.9	0.8	1.3	1.3		0.5	0.5	1.6	1.6	1.7	1.6	1.4	1.4
T. grayi (MG283141.1)	1.9	1.8	1.8	1.9	1.9	1.6	1.1	1.3	1.3	1.1	1.0	1.5	1.5	0.5		0.0	1.8	1.8	1.9	1.8	1.6	1.5
T. grayi (MG283146.1)	1.8	1.8	1.8	1.9	1.9	1.5	1.1	1.3	1.3	1.1	1.0	1.5	1.5	0.5	0.0		1.8	1.8	1.9	1.8	1.5	1.5
T. rangeli (AY230239.1)	0.9	1.1	1.1	1.2	1.2	1.1	1.0	1.2	1.2	1.5	1.5	1.4	1.4	1.6	1.8	1.8		0.1	1.0	1.0	1.4	1.4
T. rangeli (AY230238.1)	0.9	1.2	1.1	1.2	1.3	1.2	1.1	1.3	1.3	1.6	1.5	1.5	1.5	1.6	1.8	1.8	0.1		1.1	1.0	1.4	1.4
T. congolense (JN673388.1)	1.1	1.2	1.2	1.3	1.3	1.2	1.1	1.3	1.3	1.6	1.6	1.5	1.5	1.7	1.9	1.9	1.0	1.1		0.2	1.5	1.5
T. congolense (JN673389.1)	1.0	1.2	1.2	1.2	1.3	1.2	1.1	1.3	1.3	1.6	1.5	1.5	1.5	1.6	1.8	1.8	1.0	1.0	0.2		1.4	1.4
T. brucei (MK132171.1)	1.5	1.4	1.4	1.2	1.5	1.2	0.8	1.0	1.0	1.3	1.2	1.2	1.2	1.4	1.6	1.5	1.4	1.4	1.5	1.4		0.1
T. brucei (MK132169.1)	1.4	1.4	1.4	1.5	1.5	1.2	0.8	1.0	1.0	1.3	1.2	1.2	1.2	1.4	1.5	1.5	1.4	1.4	1.5	1.4	0.1	
